# Fabrication and characterization of PLGA nanoparticles encapsulating large CRISPR–Cas9 plasmid

**DOI:** 10.1186/s12951-019-0564-1

**Published:** 2020-01-20

**Authors:** Ami Jo, Veronica M. Ringel-Scaia, Dylan K. McDaniel, Cassidy A. Thomas, Rui Zhang, Judy S. Riffle, Irving C. Allen, Richey M. Davis

**Affiliations:** 1 Department of Chemical Engineering, Virginia Tech, Blacksburg, VA 24061 USA; 2 Graduate Program in Translational Biology, Medicine, and Health, Virginia Tech, Blacksburg, VA 24061 USA; 3 Department of Biomedical Sciences & Pathobiology, Virginia-Maryland College of Veterinary Medicine, Virginia Tech, Blacksburg, VA 24061 USA; 4 Department of Chemistry, Virginia Tech, Blacksburg, VA 24061 USA; 5 Macromolecules Innovation Institute, Virginia Tech, Blacksburg, VA 24061 USA; 6 Department of Basic Science Education, Virginia Tech Carilion School of Medicine, Roanoke, VA USA

**Keywords:** Nanoprecipitation, Transfection, CRISPR–Cas9, PLGA nanoparticles

## Abstract

**Background:**

The clustered regularly interspaced short palindromic repeats (CRISPR) and Cas9 protein system is a revolutionary tool for gene therapy. Despite promising reports of the utility of CRISPR–Cas9 for in vivo gene editing, a principal problem in implementing this new process is delivery of high molecular weight DNA into cells.

**Results:**

Using poly(lactic-*co*-glycolic acid) (PLGA), a nanoparticle carrier was designed to deliver a model CRISPR–Cas9 plasmid into primary bone marrow derived macrophages. The engineered PLGA-based carriers were approximately 160 nm and fluorescently labeled by encapsulation of the fluorophore 6,13-bis(triisopropylsilylethynyl) pentacene (TIPS pentacene). An amine-end capped PLGA encapsulated 1.6 wt% DNA, with an encapsulation efficiency of 80%. Release studies revealed that most of the DNA was released within the first 24 h and corresponded to ~ 2–3 plasmid copies released per nanoparticle. In vitro experiments conducted with murine bone marrow derived macrophages demonstrated that after 24 h of treatment with the PLGA-encapsulated CRISPR plasmids, the majority of cells were positive for TIPS pentacene and the protein Cas9 was detectable within the cells.

**Conclusions:**

In this work, plasmids for the CRISPR–Cas9 system were encapsulated in nanoparticles comprised of PLGA and were shown to induce expression of bacterial Cas9 in murine bone marrow derived macrophages in vitro. These results suggest that this nanoparticle-based plasmid delivery method can be effective for future in vivo applications of the CRISPR–Cas9 system.

## Background

The clustered regularly interspaced short palindromic repeats (CRISPR)–Cas9 system has received much attention recently due to its potential for revolutionizing targeted genome editing with unprecedented precision and control. CRISPR was discovered in bacteria and function as a defense mechanism against invading viral DNA [1]. Over the last two decades, CRISPR–Cas9 based systems have revolutionized our ability to rapidly and effectively target genes in eukaryotic cells for genetic modification. In biomedical research applications, CRISPR–Cas9 is now routinely utilized to generate novel genetically modified animal models and is being aggressively pursued in gene therapy applications. Indeed, a series of high profile proof-of-concept studies recently demonstrated that the CRISPR–Cas9 system could be harnessed to modify the mouse genome in adult animals and modulate disease phenotypes [2–4].

The CRISPR–Cas9 system is typically delivered to cells as a single large plasmid or multiple smaller plasmids that encode a target sequence, a CRISPR guide, and Cas9. However, efficient transfection of DNA or RNA into the cell for transcription is a significant hurdle. Both chemically- and mechanically-based transfection methods have been successfully utilized in vitro, but effective, clinically relevant, in vivo transfection methods are significantly lacking. Lipofectamine is a cationic lipid-based transfection agent often used to increase permeability of the cell membrane, but it can destabilize the membrane and result in unacceptable toxicity, especially in test animals [5]. Electroporation and sonoporation have also been examined in vivo [6, 7]. However, these approaches are not always feasible when attempting to transfect animals or humans due to accessibility limitations and invasiveness of the treatments. Finally, adenoviral vectors are also being studied as potential carriers for the CRISPR–Cas9 system and have shown great success in vitro [8, 9] yet the relatively large size of the CRISPR–Cas9 system and potential immunogenicity of adenoviral vectors have, for the moment, limited in vivo applications.

The lack of an efficient and clinically relevant delivery system is a major hurdle in advancing CRISPR–Cas9 from proof-of-principle to in vivo clinical application. Specifically, the delivery system must be clinically relevant, capable of being targeted to specific cells of interest and minimize immune system stimulation. After considering a range of delivery approaches, we believe that polymeric nanoparticles offer a promising solution to this limitation. For example, Cohen et al. [10] studied sustained marker gene expression using plasmid DNA in PLGA nanoparticles and liposomes, which concluded that while nanoparticles resulted in a much lower level of gene transfection in vitro, it produced almost two orders of magnitude more successful transfection in vivo than with liposomes. By using a polymeric nanocarrier, the bioavailability of the therapy is increased by reducing the premature clearance of these biomaterials from the body. In theory, a nanoparticle can be designed to enter the cell by endocytosis and release the plasmid directly into the cytoplasm. Poly(lactic-*co*-glycolic acid) (PLGA) has proven very useful for drug delivery given its biocompatibility, biodegradability, and toxicologically safe degradation products [11]. PLGA has been approved by the FDA for human use in nanomedicine formulations [12, 13]. Proteins, peptides, genes, vaccines, antigens, and human growth factors have been successfully incorporated into PLGA or PLGA-based particles [11]. However, to our knowledge, the use of biodegradable polymer nanoparticles to deliver plasmid DNA instead of cationic lipid condensing agents or viruses for implementing CRISPR–Cas9 gene editing has not previously been demonstrated.

This study explores the engineering and processing steps to fabricate high molecular weight plasmid DNA-encapsulated fluorescently-labeled, pegylated PLGA nanoparticles that could be used in future in vivo systems. The particles themselves are internalized by the cell and tracking is enabled by a novel fluorescent dye. For these initial proof-of-concept studies, we utilized in vitro assays with mouse bone marrow derived macrophages (BMDMs). The most relevant study prior to this work was that of Niu et al. [14] who used a modified slow nanoprecipitation method to encapsulate plasmid DNA into PLGA particles for enhanced expression of green fluorescent protein (GFP) in cells. They tested loading, structural integrity, DNA protection from enzymes when in the particles, and functionality in cell studies. This nanoprecipitation method became the starting point for the work presented here for encapsulating CRISPR–Cas9 plasmid. The main differences between the work by Niu et al. and this work was the size of the CRISPR–Cas9 plasmid (~ 8500 bp), which is approximately twice the size of their GFP plasmid (~ 4000 bp), and the need for fluorescently labeled particles to track the NP for future in vitro and in vivo applications. Because high molecular weight plasmid DNA tends to be susceptible to shear degradation, we used this low-shear nanoprecipitation method to address these particle design constraints. Nanoprecipitation forms particles by adding a water-miscible organic solution of a polymer and therapeutic drop-wise into an aqueous solution containing a polymeric surfactant which, in the work of Niu et al. [14] was the triblock copolymer Pluronic F127™. Using this modified nanoprecipitation method, we characterized fluorescently labeled PLGA nanoparticles encapsulating a high molecular weight CRISPR–Cas9 plasmid DNA and investigated their transfection in vitro in murine bone-marrow derived macrophages.

## Results

### Solvent mixture used in particle formation

**Fig. 1:**
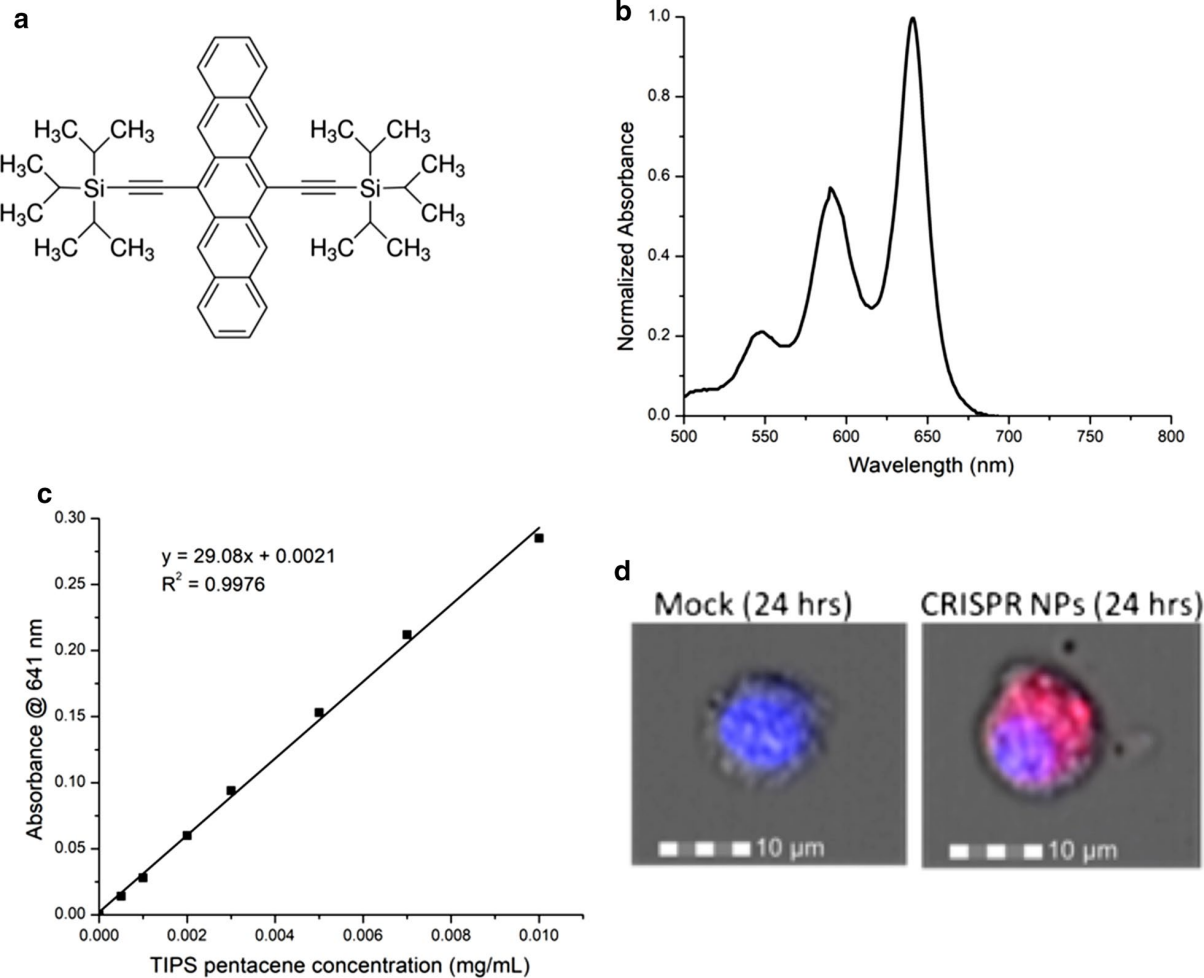
TIPS pentacene can be used as a fluorescent marker for cell internalization. **a** TIPS structure, **b** normalized absorbance spectrum of TIPS pentacene in THF (v/v), **c** calibration curve of absorbance vs. TIPS pentacene concentration in THF by volume, **d** representative imaging flow cytometer pictures selected randomly out of 10,000 BMDM cells treated with either mock or TIPS loaded NPs demonstrates internalization of particles into the cell as indicated by red fluorescence signal

Since 6,13-bis(triisopropylsilylethynyl) pentacene (TIPS pentacene), DNA, and PLGA have very different solubility characteristics, a solvent mixture was needed to solubilize all three components and form well-defined nanoparticles when mixed with the aqueous Pluronic F-127 solution. TIPS pentacene is highly nonpolar and therefore readily soluble in THF while only partially soluble in DMF (Fig. 1a, b). In contrast to TIPS pentacene, PLGA was more soluble in DMF than THF. However, DNA was not soluble in this purely organic mixture and thus a mixed aqueous-organic mixture was sought. Ke et al. [15] used a mixture of 5 vol% TE buffer and 95 vol% DMF and showed that plasmid DNA was stable in this mixture at room temperature. Therefore, to solubilize the DNA, some TE buffer was added to the mixture of DMF and THF but the concentration was kept low to prevent the TIPS pentacene from precipitating. Because the CRISPR plasmid used in this work was about twice the size as that used by Ke et al., 5 vol% was not sufficient to solubilize the DNA and was found experimentally to leave a small visible pellet of DNA that was not loaded into the syringe for particle formation. The final solvent mixture solubilizing TIPS pentacene, DNA, and PLGA consisted of 10 vol% TE buffer, 45 vol% DMF, and 45 vol% THF and was used in the nanoprecipitation process to form nanoparticles.

### Evaluation of nanoparticles made with different PLGA end groups show main difference to be in DNA loading

For particles made with the ester-end capped PLGA, the intensity-average hydrodynamic diameters (D_i_) were ~ 160 nm while the diameters of particles made with the amine-end capped PLGA were slightly larger, D_i_ ~ 180 nm with no significant change when DNA was encapsulated (Table 1). Because PLGA degrades by hydrolysis, the particles were freeze dried for extended storage which greatly simplified the subsequent cell-based experiments. Since PLGA aggregates irreversibly during freeze-drying, trehalose was used as a cryoprotectant. A mass ratio of NP to trehalose ranging from 1:25 to 1:42—determined by experiments described in Additional file 1—resulted in particles redispersed in DI water that were somewhat aggregated during freeze drying with D_i_ ~ 210–350 nm but still small enough to be useful for cell uptake. The trehalose:NP ratio varied slightly from batch to batch as the exact concentration of the final suspension after the centrifugal wash during particle fabrication varied and was calculated after the freeze-drying process. The zeta potentials of the particles showed little variation, ranging from − 29 to − 35 mV, due most likely to carboxylate groups on the particles formed due to hydrolysis of the PLGA.

**Table 1:** Size and zeta potential of NPs in DI water made with different end-capped PLGA before and after lyophilization

Sample	PLGA end cap	Target DNA loading (wt% based on PLGA)	Actual DNA loading (wt% based on PLGA)	Before freeze drying			Freeze dried 1:~ 25 NP:trehalose (w:w)		
				D_I_ (nm)	PDI	ZP (mV)	D_I_ (nm)	PDI	ZP (mV)
A	Ester (neutral)	**–**	**–**	157	0.06	− 31	344	0.42	− 29
B		2	0.7	160	0.11	− 33	213	0.23	− 35
C	Amine (+)	**–**	**–**	177	0.11	− 24	290	0.37	− 27
D		2	1.6	182	0.08	− 26	260	0.21	− 30

### Proton NMR analysis can be used to estimate Pluronic F127 versus PLGA mass ratio in nanoparticles

Because Pluronic F127 was added in excess and any material not physisorbed to the surfaces of the nanoparticles during formation was removed during the centrifugation step, it was important to determine the polymer composition of the final nanoparticle product. By determining the mass content of F127 and PLGA in the nanoparticles, the encapsulation efficiency can also be more accurately calculated. Using proton NMR, solutions of the PLGA and F127 in deuterated chloroform (CDCl_3_) separately were first analyzed.

**Fig. 2:**
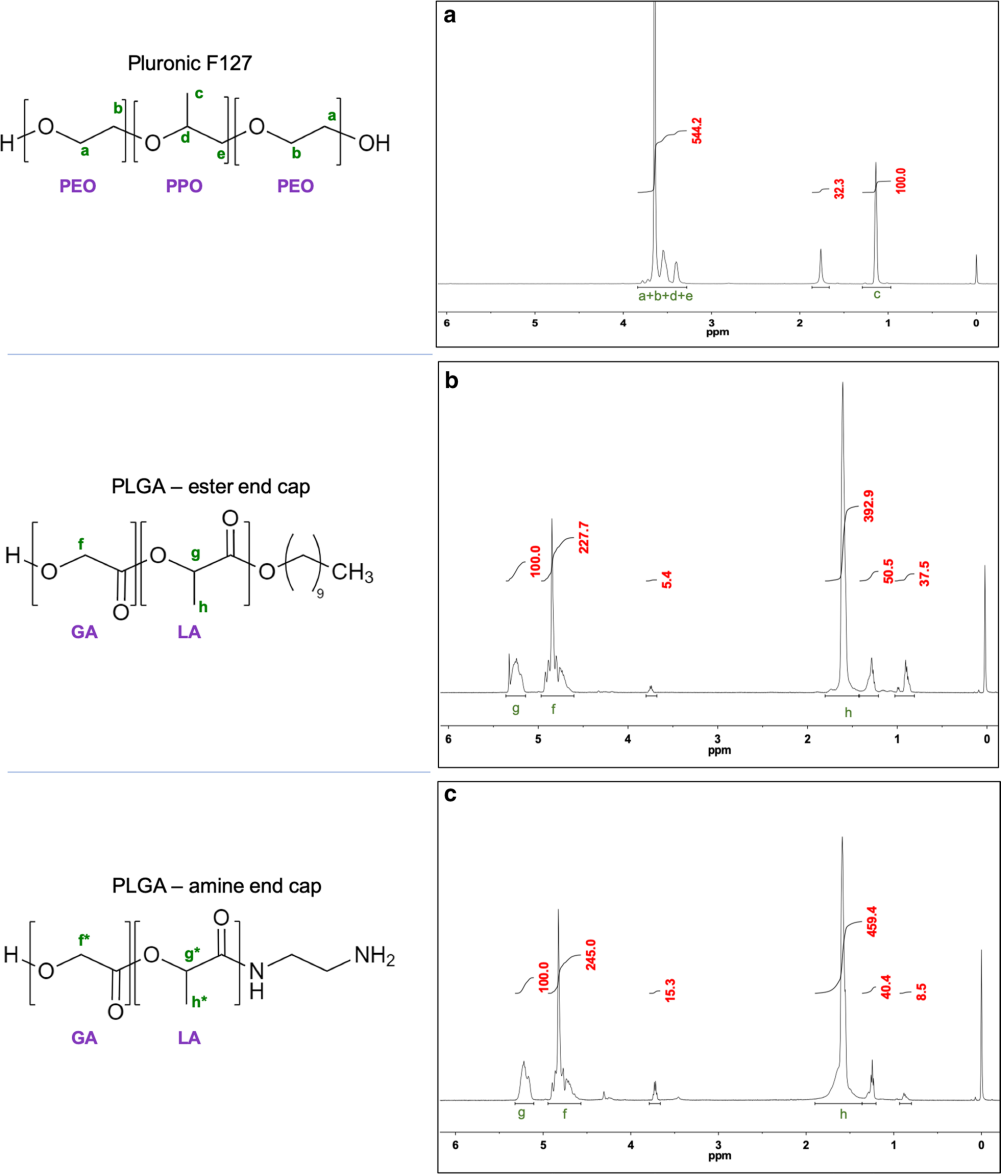
Proton NMR of materials making up nanoparticles. Analysis of F127 and PLGA proton NMR spectra show distinct peaks that can be used to determine polymer composition of resulting nanoparticles. ^1^H NMR spectra of **a** Pluronic F127 and **b** ester end capped PLGA **c** amine end capped PLGA in CDCl_3_

In the ^1^H NMR spectrum of Pluronic F127, the methyl protons have a chemical shift around 1 ppm (Fig. 2a). All other peaks from both PEO and PPO blocks are integrated into one peak, between 3.2 and 3.8 ppm, due to the presence of adjacent oxygen atoms. Based on the NMR, the composition of the F127 is PEO_108_-*b*-PPO_65_-*b*-PEO_108_ which is close to the theoretical values of PEO_100_-*b*-PPO_65_-*b*-PEO_100_ [16]. Alternatively, if the integrals of the methyl protons on the PPO segment are set to be 100, then there would be 100/3 = 33.3 repeating units of PO. The PEO methylene proton peaks then have an integral of 544.2 – 100 = 444.2, after subtracting the methylene and methine protons from the PO. It means there are 444.2/4 = 111 repeating units of EO, per 33.3 units of PO. The molar ratios of the EO over PO are then:

$$\frac{mole\,of\,EO}{mole\,of\,PO}=\frac{3.33 }{1}$$

Another important value was the percent of the integrals in the overlapped chemical shift region that contributed to the EO mass. The % value becomes important when calculating the PPO ratios in case overlapping occurs when mixed with the PLGA components. The PPO proton wt% can be calculated as follows.

$$\%\text{A}_{3.25-3.8\text{ppm}\text{PPO}}=\frac{444.2}{544.2}=81.2\%$$

The PLGA spectra show two distinctive peaks around 4.5–5.5 ppm (Fig. 2b, c). Specifically, the methine protons (g peak) from the poly(lactide) segment have a chemical shift of ~ 5–5.5 ppm, while the value for the methylene protons (f peak) from the poly(glycolide) segment was 4.5–5 ppm. The molar ratios of the poly(lactide) segment over poly(glycolide) can be determined by comparing the integral of g to the integral of f divided by two, as there are two protons in peak f compared to only one in peak g. The results indicated the molar ratios are ~ 1:1, close to the values provided by the manufacturer.

**Fig. 3:**
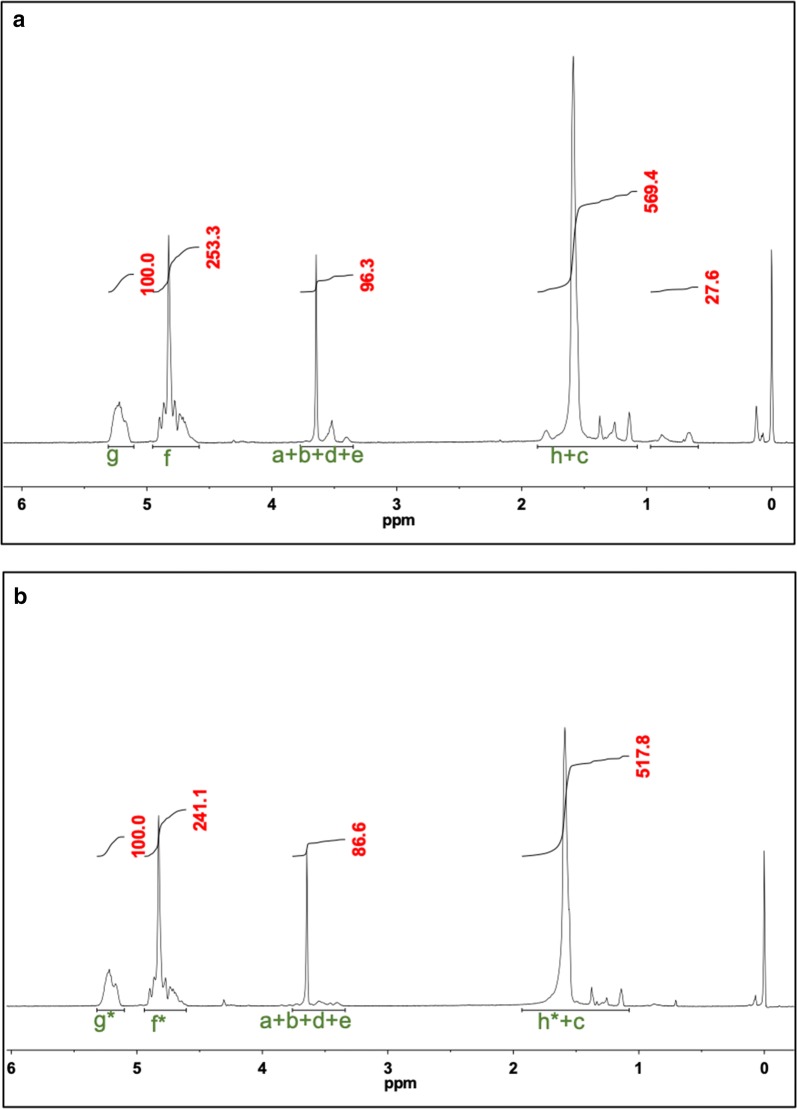
Using proton NMR to more accurately calculate DNA encapsulation efficiency. Mass ratio of Pluronic F127 and PLGA in the nanoparticles determined by proton NMR allows for more accurate calculations of encapsulation efficiency. ^1^H NMR of NPs made with Pluronic F-127 and **a** with ester end capped PLGA and **b** with amine end capped PLGA

Because the F127 and the poly(lactide) and poly(glycolide) have distinctive, non-overlapping peaks, the PLGA to F127 ratios in the TIPS loaded NPs could be calculated. For example, using the ester end cap PLGA case (Fig. 3a):

$$\frac{\text{mole of EO}}{\text{mole of PO}}=\frac{3.3}{1};\quad \frac{\text{mole of GA}}{\text{mole of LA}}=\frac{\frac{253.3}{2}} {\frac{100}{1}}={\frac{1.27}{1}}; \quad \%\text{A}_{3.25-3.8\text{ppm}\text{PPO}}=81.2\%$$

$$\frac{\text{mole \,of \, EO}}{\text{mole \,of \, LA}}=\frac{\frac{96.3*81.2\%}{4}}{100}=\frac{1}{5.11}=\frac{3.33 \text{EO}}{17.0 \text{LA}}$$

The result means that in the mixed systems, per 3.33 mol of EO, there would be 1 mol of PO, 17 mol of LA, and 17 * 1.27 mol of GA. With all these ratios, we can use the MW of the repeat units to find the mass ratio of F127 to PLGA and from there the wt% of each:

$$\frac{{\mathrm{m}}_{\mathrm{F}127}}{{\mathrm{m}}_{\mathrm{P}\mathrm{L}\mathrm{G}\mathrm{A}}}=\frac{3.33\,*\left(44.04\right)+58.06}{17.0\,*\left(1.27\,*\left(58.02\right)+72.04\right)}=\frac{204.7}{2477.3}\to 91.7\mathrm{\%}\, \mathrm{PLGA}/8.3\mathrm{\%}\, \mathrm{F}127$$

This same method can be used for the amine end cap case (Fig. 3b), yielding similar results of 92.4% PLGA. Given the approximate 5% error in the NMR spectra integration, the uncertainty of these calculations is ± 6–7% and therefore both of these compositions are statistically identical and assumed to be ~ 92% PLGA for all future loading calculations.

### DNA release profiles differ between NP formulations and pH conditions

From an analysis of the DNA content of the supernatant after centrifugation during the fabrication process, the DNA loading was determined to be 0.7 and 1.6 wt% for the ester and amine endcap PLGA cases, respectively (Table 1). The difference between the two formulations may be due to charge and hydrophobic interactions from the different PLGA end groups. We hypothesized that an amine end group would provide additional electrostatic attraction with the negatively charged DNA which would enhance loading. By contrast, the PLGA with the ester end cap contained a 9-carbon chain as the end group and thus the lower DNA loading with this polymer could be due to a combination of the lack of attractive charge interactions and the increased hydrophobicity of the chain ends which could interact unfavorably with the hydrophilic DNA. Based on the particle sizes and DNA loadings of the samples in Table 1, the estimated number of plasmids per NP ranged from ~ 2 to 5 copies (Additional file 1: Eq. S4).

**Fig. 4:**
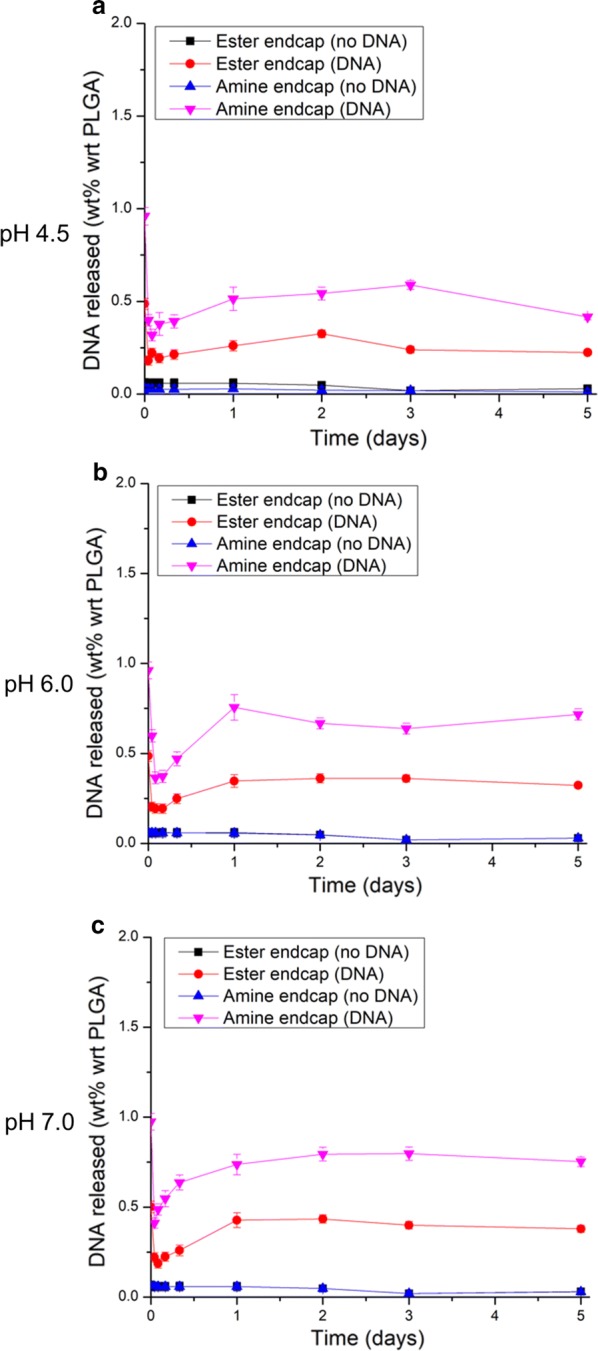
In-vitro DNA release profile from nanoparticles. Majority of DNA released within the first 24 h with NPs made from amine endcaps at pH 7 showing the highest level of release. DNA release profile from the particles with respect to time at pH **a** 7.0, **b** 6.0, **c** 4.5. % released = [mass DNA released into solution]/[initial total mass DNA encapsulated]

DNA release measurements were performed at three different pH values (pH 7, 6, 4.5) to mimic the different pH environments that the particles would experience during incubation in the media (pH 7.4) outside the cell, through early (pH 6.8–6.1) and late endocytosis (pH 6.0–4.8), and in lysosomes (pH 4.5) inside the cell [17]. The amine end-capped PLGA case shows a higher release at all three pHs, with the highest release being DNA equivalent to 0.8 wt% loading with respect to PLGA (Additional file 1: Eq. S5) at pH 7 after 3 days (Fig. 4). By contrast, the corresponding release for the ester end-capped case after 3 days at pH 7 was 0.4 wt% with somewhat lower values at pH 4.5 and 6.0. Because the amine end-capped case had over 2× higher overall loading, it is reasonable that it would release more DNA relative to the ester end-capped case.

From 1–3 days, release at pH 7.0 appeared to be systematically higher by ~ 30–60% than for the pH 4.5 case. However, it is possible that pH may affect the concentration of DNA detected by the PicoGreen assay. The first step to depurination and β-elimination during dsDNA degradation in aqueous media is catalyzed by acidic conditions [18]. Evans et al. [18] showed in their accelerated stability studies that even at a pH 6, significant difference in degradation could be seen for supercoiled plasmid DNA when compared to pH 7. The formation of acid groups due to hydrolysis is probably why lower DNA release was measured for the lower pH cases. Hydrolysis of PLGA is catalyzed by acidic conditions and, as the PLGA breaks down to form more acid groups, the local pH inside the core decreases [19]. This positive feedback loop accelerates the further breakdown of PLGA [20]. When the pH of the surrounding media was already lower as in the pH 4.5 and 6.0 cases, the acid-catalyzed hydrolysis happens more rapidly and thus could have degraded more DNA than in the more neutral pH 7 case. If the DNA is exposed to these highly acidic conditions for a long period of time, it could degrade quickly and fall below the detection limit of the assay. Balmert et al. [19] estimated the intraparticle pH of ester endcapped PLGA microparticles (MW = 15 kDa) as ~ 3–4 within 1–3 days in neutral pH media conditions. This may account for the relatively rapid release of the DNA. The particles are rapidly being hydrolyzed which, in turn, forms more acid groups that further catalyze hydrolysis, leading to formation of pores that lead to faster diffusion of DNA out of the particle into the aqueous media. However, at lower pH values, especially at pH 4.5, the acidic environment in the nanoparticles may lead to early DNA degradation, thus lowering the apparent release levels. This is supported by the decline in DNA release for the pH 4.5 case after 3 days.

The apparently anomalous points in the release profile at the t = 0 time point at all three pH values occurred when the buffer was initially added to the NPs, followed by immediate centrifugation to obtain the supernatant for the PicoGreen assay. The DNA content at this time point was found to be higher than the subsequent 1-h time point for all six of the DNA-containing conditions tested. We believe this was due to surface-bound or partially encapsulated DNA that may have been partly degraded during processing due to its exposure to the environment. Because that DNA was close to the surface, it was quickly released. Although all processing steps were done carefully to minimize degradation, between mixing, centrifugation, freeze drying, and reconstitution in buffers, it is possible that some of the surface-bound DNA had degraded. We hypothesize that, at t = 0, this surface-bound DNA was released rapidly and detected by the assay. If this DNA had already been partially degraded to form relatively short linear DNA fragments due to the effects of the handling steps, it may have degraded faster once in the media with its chain length eventually falling below the detection limit of PicoGreen (< 200 bp) as specified by the manufacturer. Other sources have shown experimentally that PicoGreen could accurately detect DNA chains as short as 150 bp [21, 22]. Regardless of the cutoff length for detection, the hypothesis of partially degraded DNA chains on the surface undergoing rapid burst release and degradation to lengths below detection by PicoGreen still applies.

This degraded DNA can also show up as a stronger signal for the same amount of DNA than when in plasmid form given the nature of the PicoGreen assay. The assay involves intercalation of the reagents into the DNA and therefore will not have access to the entire chain when the plasmid is supercoiled. Holden et al. [23] reported that, for their plasmid, the PicoGreen assay showed the supercoiled plasmid to be 60% the mass of the same plasmid that had been linearized. The discrepancy between the supercoiled and linearized forms will depend on the sequence and conformation of the plasmid but, in all cases, the supercoiled case may show a lower signal due to inaccessibility of parts of the chain. The DNA concentration for the stock solution was measured by UV absorption using a NanoDrop 2000 (ThermoFisher) which is thought to be more accurate than the PicoGreen assay for plasmids. DNA concentration measurements made with the NanoDrop 2000 were used to concentrate the stock for a targeted 2 wt% DNA loading with respect to mass of PLGA. Under the assumption that the added DNA was enough for exactly 2 wt% DNA loading, the unincorporated DNA and encapsulated DNA should add up to that total mass added. However, given the lowered detection by PicoGreen, the mass of unincorporated DNA as measured from the supernatant using PicoGreen would be an underestimate. Similarly, the DNA released over the 5 days was ~ 50% of what was loaded. These measurements are also underestimates and could be a main factor in accounting for the missing mass in the mass balance.

More important than the actual estimated loading is the DNA released as shown by the release study. The amount of measured DNA released for the amine case after 5 days at pH 7.0 was equivalent to a DNA loading of 0.8 wt% with respect to PLGA or approximately half of the total 1.6 wt% loading. This corresponds to ~ 2–3 plasmid copies released per NP and is a rough underestimate as mentioned above. An underestimated plasmid release is better than an overestimate in this application because the chances of successful delivery of the plasmid to the nucleus for transcription increases with the number of plasmid copies released. Therefore, the particles may be more effective for the apparent DNA added. To test this, cell studies were conducted to investigate the expression of the Cas9 protein and to explore any changes to the mouse DNA after NP treatments.

### Bacterial *S. pyogenes* Cas9 protein is successfully translated inside murine macrophages

**Fig. 5:**
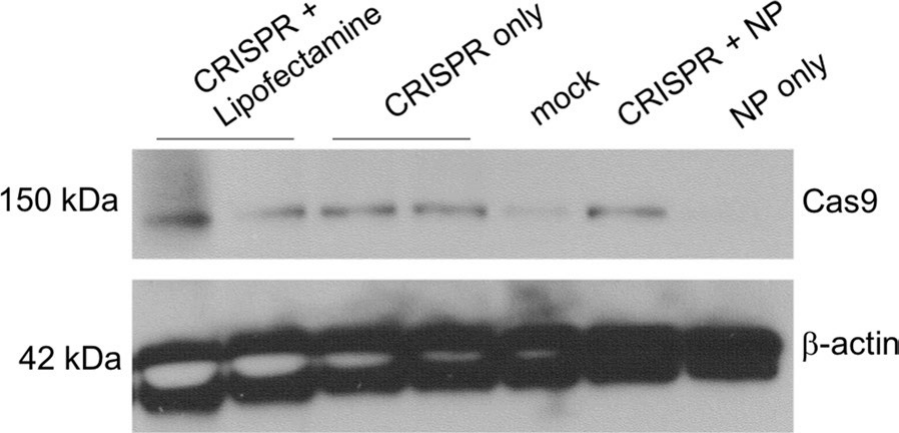
Bacterial *S. pyogenes* Cas9 protein is successfully translated inside murine macrophages. After 24 h incubation with CRISPR+ Lipofectamine (lanes 1 and 2), CRISPR plasmid only (lanes 3 and 4), PBS only (lane 5), CRISPR loaded nanoparticle (lane 6), and blank nanoparticle (lane 7), *S. pyogenes* Cas9 protein was detectable by Western Blot

To further test the successful encapsulation of CRISPR plasmid into the amine end-capped PLGA nanoparticles, we next wanted to determine whether the plasmid remained functional, defined by its ability to transcribe and translate *S. pyogenes* Cas9 protein. To do so, we harvested wild type mouse bone marrow derived macrophages (BMDMs), replated at a density of 500,000 cells/mL, and challenged the macrophages with either blank nanoparticles (100 µg/mL), CRISPR plasmid-loaded nanoparticles (100 µg/mL), CRISPR plasmid with Lipofectamine 3000 transfection (2 µg/mL DNA), CRISPR plasmid only (2 µg/mL), or PBS for 24 h. The total remaining cells were removed from the plates, lysed, and Western blot was performed for Cas9 using a *S. pyogenes* specific monoclonal antibody (Fig. 5). BMDM’s will readily phagocytose plasmids, such as the CRISPR–Cas9 plasmid used here, and Lipofectamine is a common method for plasmid transfection. Thus, we anticipate that both of these methods will successfully introduce the functional CRISPR plasmid into our BMDMs under our experimental conditions. However, neither of these methods are appropriate for in vivo use where Lipofectamine has well known toxicity issues and is not biocompatible. Likewise, the naked plasmid lacks stability and is rapidly degraded once administered in vivo. The goal of these studies is to compare the functionality of the plasmid following nanoparticle delivery against these more typical approaches.

The nanoparticle concentrations were chosen to keep the DNA concentration constant between the samples under the assumption of 2 wt% targeted DNA loading. However, the measured loading was 1.6 wt% with respect to the PLGA, and with the presence of F127 included in the total NP mass, the nominal DNA concentration of the plasmid NP case was approximately 1.5 µg/ml. In order to control for suboptimal nanoparticle delivery of CRISPR plasmids, we used Lipofectamine 3000 (Invitrogen) in order to transfect approximately the same total DNA that was encapsulated in the particles. Due to the phagocytic nature of the BMDM primary cells that we used for this study, we also treated the cells with the free plasmid DNA. Cas9 was detectable in the cells transfected with Lipofectamine (lanes 1 and 2) as well as the cells treated with CRISPR plasmid only (lanes 3 and 4) and CRISPR-loaded nanoparticle (lane 6), while the cells treated with blank nanoparticle (lane 7) and PBS only (lane 5) were not (Fig. 5). Qualitatively, the band intensities between all three CRISPR-containing samples were comparable. Again, given the phagocytic nature of these cells, the BMDMs internalized the plasmid-only control with no additional carrier or transfection needed. From release studies shown earlier, we showed that most of the plasmid was released from the particles within the first 24 h in suspension, and more specifically within the first 8 h. From imaging cytometry, we counted 10,000 cells per experimental condition and found 95% of the macrophages treated with nanoparticles exhibited red fluorescence from the TIPS pentacene indicating internalization after 24 h (Fig. 1d). McDaniel et al. showed similar statistics using TIPS pentacene loaded poly(lactic acid)-based nanoparticles. That study also showed that within the first 2 h ~ 30% of cells showed particle uptake increasing to ~ 40% at 4 h but not reaching the 90+% until after 8 h of incubation [24]. Assuming similar DNA release kinetics in cell culture media, and similar particle uptake behaviors with these PLGA particles, it is difficult to discern whether the entire nanoparticle was internalized by the macrophages before releasing the plasmid into the cytosol as intended, the plasmid in the particles were released outside the cell and the free plasmids phagocytosed, or a combination of the two. We hypothesize this will become clearer in future in vivo studies. Cohen et al. [10] found that nanoparticles performed better than liposomes for in vivo delivery of plasmid DNA for gene editing applications, although it did not do as well in in vitro cell studies. Even though we cannot see a clear advantage in using transfecting agents from this particular study, what this result does show is that the encapsulated high molecular weight plasmids in the nanoparticles were intact enough to express the Cas9 protein and can therefore be considered functional. In the current set of studies, we cloned a test gRNA targeting the Lps-d allele in the mouse *Tlr4* gene into our pX330 CRISPR plasmid [25]. Future studies will include functional and validated CRISPR gRNAs that target a range of murine genes of interest both in vitro and in vivo.

## Discussion

CRISPR–Cas9 is an extremely valuable tool towards an effective therapy for a vast range of genetic diseases. Successful delivery of high molecular weight plasmid DNA into cells is a significant hurdle in the application of CRISPR/Cas9 based therapeutic strategies. With most of the current methods for transfection being ex vivo, there is a clear need for investigations into other delivery methods. By using polymer nanoparticles, the CRISPR–Cas9 tool can be translated to in vivo therapies without the immunogenicity concerns of viral vectors or cationic liposomes. This proof-of-concept study has shown a method of fabricating versatile particles made from biocompatible materials that can be applied to multiple CRISPR–Cas9 systems and beyond to other plasmid-based treatments. The particles were shown to release 2–3 plasmid copies per particle with loadings as high as 1.6 wt% with respect to PLGA when previous studies using this method of fabrication have encapsulated plasmids half the size only at 1 wt% [14].

Previous studies have shown promising results using adenoviral vectors but historically, viral vectors have had low success rates in FDA approval [26]. However, there have been major recent steps in the development of liposomes and lipid nanoparticles to deliver CRISPR–Cas9. Researchers have used gold nanoparticles coated with lipid layers to passivate the surface and also to encapsulate the Cas9-gRNA ribonucleoprotein and donor DNA. Lee et al. [27] used gold nanoparticles due to the ability to coat a thick layer of DNA on the surface and their tendency to be internalized by many different cell types. Wang et al. [28] used gold nanoparticles as a thermally triggered release mechanism for plasmid-loaded gold nanoparticle and lipid complexes. Finn et al. [29] used lipid nanoparticles to encapsulate mRNA/gRNA complexes and demonstrated delivery in vivo. There are advantages and disadvantages to using the different forms of CRISPR–Cas9 and different delivery vehicles.

By using plasmid DNA, we are able to deliver a high Cas9 dosage with indefinite production by the cells, but there are higher risks for off-target effects [30]. One disadvantage of liposomal and lipid nanoparticle formulations is that the product is difficult to characterize and store. The liposomes are made in solution so the product yield and encapsulation are characterized solely on the payload concentration. The initial molar ratios of the different lipids and components are given under the assumption that the end product has the exact same ratios. It is difficult to determine the absolute loading of the payload and long-term storage can be an obstacle. By using polymeric nanoparticles, the particles can be freeze-dried to increase shelf-life, the material composition of the particles can be characterized to determine loading so that dosages can be identified by product mass concentrations, and the particles are dispersible at desired concentrations. Without much literature on the direct comparison of liposomal delivery versus polymer nanoparticle delivery in vivo, it is difficult to give a clear functional advantage of one over the other. However, future work in this work will include a comparison especially given the larger number of studies showing successful implementation of liposomal/lipid nanoparticle CRISPR–Cas9.

Several studies that have successfully employed nanoparticle delivery of CRISPR–Cas9 plasmids have done so using immortalized cell lines [31–33]. In this way, our study is unique as we utilize primary cells. There has been a previous study by Jin et al. [33] using a magnetic nanoparticle delivery system to transfect rat primary cortical neurons with a CRISPR plasmid, but delivery to these primary cells were found to be at a much lesser degree relative to studies done in immortalized HEK cells. Therefore, delivery in primary cell systems is an needed area of investigation in this field. With the ultimate goal of applying this method of CRISPR-loaded nanoparticles in vivo, primary cells are advantageous as they more accurately represent physiological conditions.

With multiple plasmid copies per particle, the nanoparticle-based carrier described here successfully delivered the high molecular weight CRISPR/Cas9 plasmid into primary mouse derived macrophages. The expression and translation of the bacterial Cas9 protein in NP delivered plasmids at levels comparable to cells transfected using more traditional approaches, such as Lipofectamine, was lower for the particle cases. However, this can be attributed to the 80% encapsulation efficiency of the DNA. Previous studies have shown that polymer carrier systems can be much more effective at delivering genetic material in vivo even when levels of transfection are less than levels observed in cell studies [10]. Thus, given the successes of this formulation in vitro, future work involving transfer of this technology into in vivo animal studies for comparison to current methods of delivery looks promising. It is our hope the procedures described here will ultimately improve genome editing as a whole to move us towards an effective treatment for a range of genetic diseases.

## Conclusions

The CRISPR–Cas9 gene editing system has enormous potential for the targeted editing of genes in animals but current methods for delivering plasmids for CRISPR–Cas9 system to animals using viral vectors or cationic lipid condensing agent have some drawbacks. In this work, plasmids for the CRISPR–Cas9 system were encapsulated in nanoparticles comprised of PLGA stabilized with a Pluronics triblock copolymer and containing a fluorescent marker using a simple precipitation procedure. The nanoparticles were incubated with murine bone marrow derived macrophages in vitro and, after 24 h, approximately 95% of the macrophages had internalized the particles. These macrophages expressed bacterial Cas9, which suggests that this nanoparticle-based plasmid delivery method can be effective for future in vivo applications of the CRISPR–Cas9 system.

## Material and methods

### Materials

PLGA AP063 (15,000–25,000 MW advertised (31,700 MW by GPC), PDI = 1.86, 50:50 lactic acid:glycolic acid, ester end-capped), and PLGA AI063 (10,000–20,000 MW advertised (16,000 MW by GPC), PDI = 1.41, 50:50 lactic acid:glycolic acid, diamine end-capped) from Akina Inc. PolySciTech was used as purchased. Pluronic^®^ F127 copolymer (PEO(~ 4.3 kDa)-PPO(~ 3.9 kDa)-PEO(~ 4.3 kDa)), d-( +)- Trehalose dehydrate, uninhibited tetrahydrofuran (THF) (anhydrous ≥ 99.9%), dimethyl sulfoxide (DMSO) (≥ 99.9%) and 6,13-bis(triisopropylsilylethynyl)pentacene (TIPS pentacene) were purchased from Sigma Aldrich and used as received. Dimethylformamide (DMF) (spectrophotometric grade), and chloroform (HPLC grade) was purchased and used as received from Spectrum Chemical. Deuterated chloroform (CDCl_3_) (D, 99.9%) were purchased from Cambridge Isotope Laboratories, Inc. and used as received. GE Hyclone Phosphate Buffered Saline (1×, 0.0067 M PO_4_, without Calcium and Magnesium) (PBS), Tris-Ethylenediaminetetraacetic acid (Tris-EDTA or TE) buffer, and PicoGreen Assay Kit were purchased from Fisher Scientific and used as received. CRISPR Cas-9 DNA (~ 8500 bp; MW ~ 5.5 × 10^6^ g/mol) was cultured using *Escherichia* coli (described below) and purified using the Qiagen plasmid purification maxi prep kit. Autoclaved Milli-Q deionized water (resistivity ~ 18 MΩ-cm produced from a Millipore Synergy Ultrapure Water system) was used in all experiments.

### CRISPR plasmid design

The CRISPR plasmid pX330-U6-Chimeric_BB-CBh-hSpCas9 was a gift from Feng Zheng (Addgene plasmid # 42230) [34]. The plasmid was digested with the restriction enzyme BbsI, and a murine *Tlr4*-specific gRNA was cloned into the vector. The goal with the design of the gRNA was to specifically target site 2342 in the *Mus musculus Lps-d* allele. The oligos were designed based on this target sequence, self-complimentary, and with overhang specified in order to be successfully cloned into the pX330 plasmid once digested with BbsI restriction enzyme. The sequences of the oligos in the 5′ to 3′ direction: CACCCCTGGTG TAGCCATTGCTGCCAA and AAACTTGGCAGCAATGGCTACACCAGG (Additional file 1: Fig. S1).

Competent *E. coli* cells (Sigma Aldrich) were transformed with cloned pX330 containing *Tlr4* gRNA and amplified in Ampicillin-containing selection media according to standard transformation protocols. Plasmid DNA was isolated from competent cells via plasmid MaxiPrep kit (QIAgen) following the manufacturer’s protocol, and the concentration was verified by a NanoDrop 2000 (ThermoFisher).

### Fabrication of DNA loaded PLGA nanoparticles by nanoprecipitation

The method used for nanoprecipitation of PLGA nanoparticles was modified from that previously described by Niu et al. [14] PLGA with two different end groups (ester and amine groups) were used to test the hypothesis that the positively charged amine end caps could increase the encapsulation efficiency and loading due to the charge interactions between it and the negatively charged backbone of the DNA. In a 50 mL polypropylene conical centrifuge tube, 100 mg Pluronic F127 was dissolved in 20 mL autoclaved DI water by vortex mixing followed by 30 min of sonication (Fisherbrand CPXH Series Heated Ultrasonic Cleaning Bath; 110 W, 40 kHz). An autoclaved magnetic stirring bar was added and the solution was mixed at 600 RPM for 30 min while the other solutions were made. Plastic labware was used instead of glassware throughout to minimize nonspecific adsorption of DNA. Solutions of PLGA dissolved in DMF (44.48 mg/ml) and TIPS pentacene dissolved in THF (0.667 mg/ml) were made separately. The PLGA was left quiescently to wet in DMF for 30 min before being sonicated for 30 min but the TIPS pentacene was only vortex mixed and not sonicated to avoid degradation from heat and sonication. Next, 400 µL of a 1.41 mg/mL stock solution of plasmid DNA in TE buffer were placed in an Amicon 100 kDa MWCO Ultra 0.5 centrifugal filter and spun at 11 k RPM (11,498×*g*) (Fisher Scientific accuSpin™ Micro; PCR-Rotor 7500 3243) for 10 min to concentrate the solution. The filter was then inverted into a clean tube and spun at 3 k RPM (855×*g*) for 3 min to recover the now-concentrated DNA (~ 8.14 mg/mL). Next, 63 µL of the concentrated DNA solution was diluted with 63 µL TE buffer. To make the final solution, 562 µL of PLGA stock, 562 µL TIPS pentacene stock, 126 µL TE (with or without DNA) were combined in a 1.5 mL centrifuge tube with gentle pipetting until visually homogenous. This solution had a volume of ~ 1.25 mL and contained 25 mg PLGA, 0.513 mg DNA, and 0.375 g TIPS corresponding to a 2 wt% DNA and 1.5 wt% TIPS loading with respect to the PLGA. The mixture was then loaded into a 3 mL plastic syringe fitted with a 21-gauge beveled needle. Using a syringe pump (New Era Pump Systems, Farmingdale, NY), the organic solution was added drop-wise (30 mL/h) to the aqueous Pluronic F-127 solution magnetically stirred at 600 rpm. The combined solution was left stirring for 5 h on ice and covered to minimize light exposure for the TIPS pentacene before being centrifuged at 4 °C and 22,789×*g* for 30 min (Thermo Scientific, Sorvall Legend X1R, FIBER*Lite*™ F15-8×50c rotor). The supernatant was decanted and saved for assaying the unincorporated DNA by PicoGreen. The pellet was re-suspended in 20 mL of DI water by 30 min of sonication and then filtered through a 0.45 µm nitrocellulose membrane to make the “reserve” suspension. Meanwhile, 200 mg of trehalose were dissolved in 1 mL of DI water. Next, 1.5 mL of the reserve suspension was split into three 1.5 mL tubes (0.5 mL per tube) to freeze dry without a cryoprotectant to determine the concentration of the reserve suspension. The remaining ~ 16–18 mL was added to the trehalose mixture for a targeted NP:trehalose mass ratio of 1:25. All samples were frozen in a − 70 °C freezer and then lyophilized (FreeZone6, LABCONCO) for at least 5 days at < 0.09 mBar and ~ −50 °C.

### Size and zeta potential characterization

Size distributions were measured by dynamic light scattering (Zetasizer Nano-ZS, Malvern Instruments, software version 7.12) at 25 °C unless otherwise noted. The sizes reported are averages of five measurements of the intensity peak corresponding to the bulk of the mass in the system. Each measurement consisted of 12–16 sub-runs averaged by the software and all solutions had a NP concentration of ~ 0.2 mg/mL. For the measurement of reserve suspensions, 50 µL of sample were diluted with 1 mL of DI water. For solutions made with freeze-dried samples, the powdered samples were left quiescently to wet for 15 min followed by vortex mixing and then sonication for 1 min. Zeta potentials were also measured using the same suspensions used for DLS (Zetasizer Nano-ZS) which were loaded into pre-wetted folded capillary tubes. Five measurements were conducted per sample with each measurement consisting of an average from 42 sub-runs.

### Determining PLGA and Pluronic content per nanoparticle by proton NMR

Proton NMR was used in order to find the mass ratio of PLGA to Pluronic content in nanoparticles. PLGA, Pluronic, and TIPS pentacene loaded nanoparticles were dissolved separately in CDCl_3_ at concentrations of ~ 1–2 mg/mL, and placed in standard 5 mm o.d. tubes. The ^1^H NMR spectra were obtained using a Bruker Avance 500 spectrometer operating at 500 MHz and 25 °C with 32 scans per sample.

### DNA release profile

Using the approximate ratio of trehalose:NP as described in Additional file 1, ~ 9–10 mg of NPs were weighed into three separate 15 mL tubes for each of the 4 cases, i.e. the ester and the amine end-capped PLGA types with and without DNA for a total of 12 samples. The three tubes of each NP case were resuspended in PBS at three different pHs (4.5, 6.0, 7.0) at a final nanoparticle concentration of 1 mg/mL. The buffers started from 1× PBS and were titrated to the target pHs using HCl measured using a pH meter (Denver Instrument UB-5 with ThermoScientific Orion™ 9156DJWP Double Junction Electrode). The samples were left to equilibrate overnight and titrated again back to the target pH. The buffers were then autoclaved for sterility and aliquots were tested a final time for correct pH to keep the stock sterile. The 15 mL tubes of suspensions were vortexed and sonicated for 1 min before being aliquoted to nine separate 2 mL centrifuge tubes for each of the time points. The tubes were stored in a 37 °C incubator on a nutating mixer (Fisher Scientific Nutating Mixer Variable Speed 3D Platform Rotator Model # 88861043) at 15 RPM and removed at specified time points for analysis. At each time point, the particles were spun down at 16,060×*g* for 20 min at room temperature and 500 µL of the supernatant were used for the PicoGreen assay. A stock volume (1 mL per time point) of each dilution for the lambda DNA standard calibration was made to keep the standard concentrations consistent across all times points.

### Macrophage nanoparticle and CRISPR challenge

Bone marrow derived macrophages (BMDMs) were isolated from wild type mice following standard procedures [35]. Briefly, bone marrow isolated from the femur and tibia from C57Bl/6 female mice aged 8–12 weeks was incubated for 6 days with Dulbecco Modified Eagle Media (DMEM) supplemented with 10% fetal bovine serum, 1× penicillin/streptomycin, and 20% L929-conditioned media. On day 6, total cell numbers were counted and replated at a cell density of 500,000 cells/mL. After overnight incubation with complete DMEM supplemented with 10% fetal bovine serum and 1× penicillin/streptomycin, the macrophages were resuspended with plain DMEM and either blank nanoparticles (100 µg/mL), CRISPR plasmid-loaded nanoparticles (100 µg/mL), CRISPR plasmid with Lipofectamine 3000 (2 µg/mL DNA), CRISPR plasmid only (2 µg/mL), PBS for 24 h.

## Supplementary information

**Additional file 1.** Includes target sequence for CRISPR system and details into analysis of DNA encapsulation and release.

## Data Availability

All data generated or analyzed during this study are included in this published article and in additional files.

## Abbreviations

CRISPR: clustered regularly interspaced short palindromic repeats
PLGA: poly(lactic-*co*-glycolic acid)
TIPS: 6,13-bis(triisopropylsilylethynyl)
BMDM: bone marrow derived macrophages
THF: tetrahydrofuran
DMSO: dimethyl sulfoxide
DMF: dimethylformamide
TE: tris-Ethylenediaminetetraacetic acid
