# Supplementary material for: Fabrication and characterization of PLGA nanoparticles encapsulating large CRISPR–Cas9 plasmid
Source: J Nanobiotechnology. 2020 Jan 20;18:16. doi: 10.1186/s12951-019-0564-1 (PMC6970287; doi:10.1186/s12951-019-0564-1)
Supplement: Supplementary file 1 — Additional file 1. Includes target sequence for CRISPR system and details into analysis of DNA encapsulation and release. [file 12951_2019_564_MOESM1_ESM.docx]

## Supplementary Materials

**
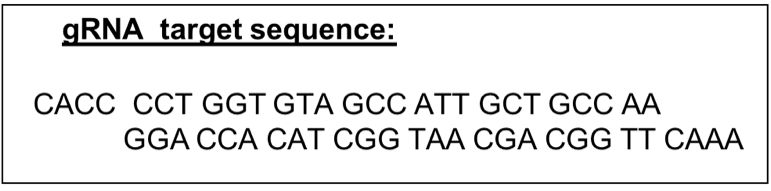
**

**Figure S1.** Designed target sequence for gRNA in CRISPR Cas9 plasmid.

###

### Measurement and calculation of TIPS pentacene encapsulation

The targeted TIPS pentacene loading of 1.5 wt% was based on the mass of TIPS pentacene and PLGA dissolved in the solvent mixture used for fabrication. As with the NMR measurements, NPs for the TIPS pentacene composition measurements were freeze dried without trehalose cryoprotectant. The freeze dried nanoparticles were then dissolved in THF at concentrations of 0.2 and 0.5 mg/mL with vortex mixing and no sonication, again to protect TIPS pentacene from thermal degradation. TIPS exhibits a local absorbance maximum at 641 nm (**Fig. 1b**) where PLGA and Pluronic F127 do not absorb at all and so the absorbance at this wavelength was used to quantify TIPS pentacene loading. The absorbances of these solutions were compared to an absorbance vs. TIPS pentacene concentration calibration curve (**Fig. 1c**) to determine the TIPS pentacene concentration in the NP solution, C_TIPS pentacene_ according to:

| $Absorbance=29.084*C_{TIPS pentacene}+0.0021$ | (S1) |
| --- | --- |

The calculated TIPS concentration was subtracted from the known NP concentration to obtain the polymer concentration in solution and then, using the PLGA/F127 mass ratio of 92/8 determined by NMR (described below), the PLGA concentration was calculated which permitted a calculation of the TIPS pentacene loading relative to the PLGA content. The loading was then used to back calculate the encapsulation efficiency defined as:

| $EE= \frac{wt\% TIPS pentacene determined by absorbance measurement}{wt\% TIPS pentacene targeted during fabrication}$ | (S2) |
| --- | --- |

For example, a 0.2 mg/mL concentration of NP dissolved in THF had an absorbance of 0.075 so that:

$$TIPS pentacene concentration=\frac{Absorbance+0.0021}{29.084}=\frac{0.075+0.0021}{29.084}=0.0025\frac{\mathrm{mg}}{\mathrm{mL}}$$

The TIPS pentacene loading, defined as (TIPS mass/PLGA mass) is given by:

$$TIPS pentacene loading=\frac{0.0025}{\left( 0.2-0.0025 \right)*92\% PLGA}x100\%=1.38 \pm0.11 wt\%$$

and the TIPS encapsulation efficiency, EE, relative to the PLGA is:

$$EE= \frac{wt\% TIPS pentacene calculated}{wt\% TIPS pentacene targeted during fabrication}=\frac{1.38 wt\%}{1.5 wt\%}=92\% EE$$

The major source of uncertainty comes from the propagation of uncertainty in the PLGA composition given the NMR measurements. This analysis shows that almost all of the TIPS pentacene was encapsulated which is consistent with the highly hydrophobic nature of the small molecule fluorophore.

*Determining NP:trehalose ratio using TIPS pentacene*

From the 3 tubes of 0.5 mL reserve suspension that were freeze dried without trehalose, the NP concentration of the reserve suspension (eq. (S3)) was determined using the TIPS pentacene calibration curve and the calculated TIPS pentacene loading.

| $C_{NP reserve}= \frac{C_{TIPS tube}}{{wt\%}_{TIPS(wrtNP)}}*100$ | (S3) |
| --- | --- |

where C_NP reserve_ is the NP concentration in the reserve suspension, C_TIP tube_ is the concentration of TIPS pentacene in the dissolved NP samples from the freeze dried tubes, and wt%_TIPS(wrtNP)_ is the wt% TIPS pentacene with respect to the entire NP (PLGA + Pluronic F127) as measured from UV-Vis spectrophotometry of a known mass of NP (not to be confused with the wt% TIPS calculated with respect to PLGA as done during the EE calculations) (wt%_TIPS(wrtNP)_ = 1.25). For Eq. (S3), it is also assumed that the freeze-dried particles were dissolved in the same volume of THF as the volume of reserve suspension added to the tube (0.5 mL).

To calculate the mass of NPs added, the reserve concentration was multiplied by the volume of suspension added to the trehalose tube. This mass was compared to the 200 mg trehalose added per batch to determine the final NP:trehalose mass ratio which ranged from 1:25 to 1:42.

### Measuring DNA loading by PicoGreen assay

To determine the DNA loading of the particles, the free DNA concentration in the supernatant that was not encapsulated into the particles was measured using the PicoGreen dsDNA assay. After the particles were centrifuged during fabrication, the supernatants were decanted into new 50 mL centrifuge tubes. The supernatant was diluted 10-fold into 1 mL aliquots for the measurement. The samples were measured as per instruction of the assay and compared to a set of lambda DNA solutions ranging 0-2000 ng/mL. The assay was read on black 96-well plates using a plate reader (BioTek, SynergyMx) with the sensitivity adjusted so that the 2000 ng/mL signal was below the saturation level of the detector. The DNA concentration of the supernatant was back calculated assuming 21 mL volume (20 mL F127 solution + 1 mL organic solution) and 0.41 mg DNA added in the 1 mL organic during particle formation.

### Estimation of the number of plasmid copies per particle

Estimating the number of plasmid copies per particle starts with the hydrodynamic diameter as measured by DLS. The individual PEG chains from the Pluronic F127 that coat the surface of the particles have a molecular weight of ~ 4400 g/mole and are assumed to be ~15 nm as assumed by Pansare et al. in their own fluorescence per nanoparticle calculations.^33^ With the known wt% DNA with respect to PLGA as measured by picoGreen and NMR, we can calculate the approximate plasmid copies per particle with the following equation:

| $\frac{\#plasmids}{\mathrm{NP}}= \frac{\pi{{(d}_{\mathrm{core}})}^{3}\rho_{\mathrm{PLGA}}*{wt\%}_{\mathrm{DNA}}*N_{A}}{\mathrm{MW}_{\mathrm{DNA}}}$ | (S4) |
| --- | --- |

where d_core_ is the estimated diameter of the hydrophobic PLGA core [cm], ρ_PLGA_ is the density of bulk PLGA [1.34 g/cm^3^]^34^, wt%_DNA_ is the measured loading of DNA with respect to PLGA, N_A­­_ is Avogadro’s number, and MW_DNA­_ is the molecular mass of the 8500 bp plasmid [g/mol] assuming 650 g/mol base pair.

### Calculations of DNA loading with respect to PLGA

The wt% DNA with respect to PLGA values that make up the y-axis for **Fig. 5** in the main text was calculated from picoGreen assays of the DNA concentrations in the supernatant during the release studies at known NP concentrations.

| ${wt\%}_{\mathrm{DNA}}= \frac{C_{DNA picogreen}}{C_{\mathrm{NP}}*{wt\%}_{\mathrm{PLGA}}}$ | (S5) |
| --- | --- |

where C_DNA picogreen_ is the DNA concentration as measured by the picoGreen assay, C_NP_ is the known NP concentration in the suspension, and wt%_PLGA_ is the percent PLGA that makes up the polymer carrier as determined by NMR analysis. The mass of DNA is not accounted for in the total mass calculation of the denominator because the DNA loadings with respect to the entire nanoparticle is less than 1 wt% and considered negligible.
